# Supplementary material for: Association between oral microbiome and seven types of cancers in East Asian population: a two-sample Mendelian randomization analysis
Source: Front Mol Biosci. 2023 Nov 21;10:1327893. doi: 10.3389/fmolb.2023.1327893 (PMC10702768; doi:10.3389/fmolb.2023.1327893)
Supplement: Supplementary file 1 [file DataSheet1.ZIP › Supplementary Materials/MR plots of saliva/saliva═╝/Lung Cancer/pheno.1139_to_lung cancer_forest.pdf]

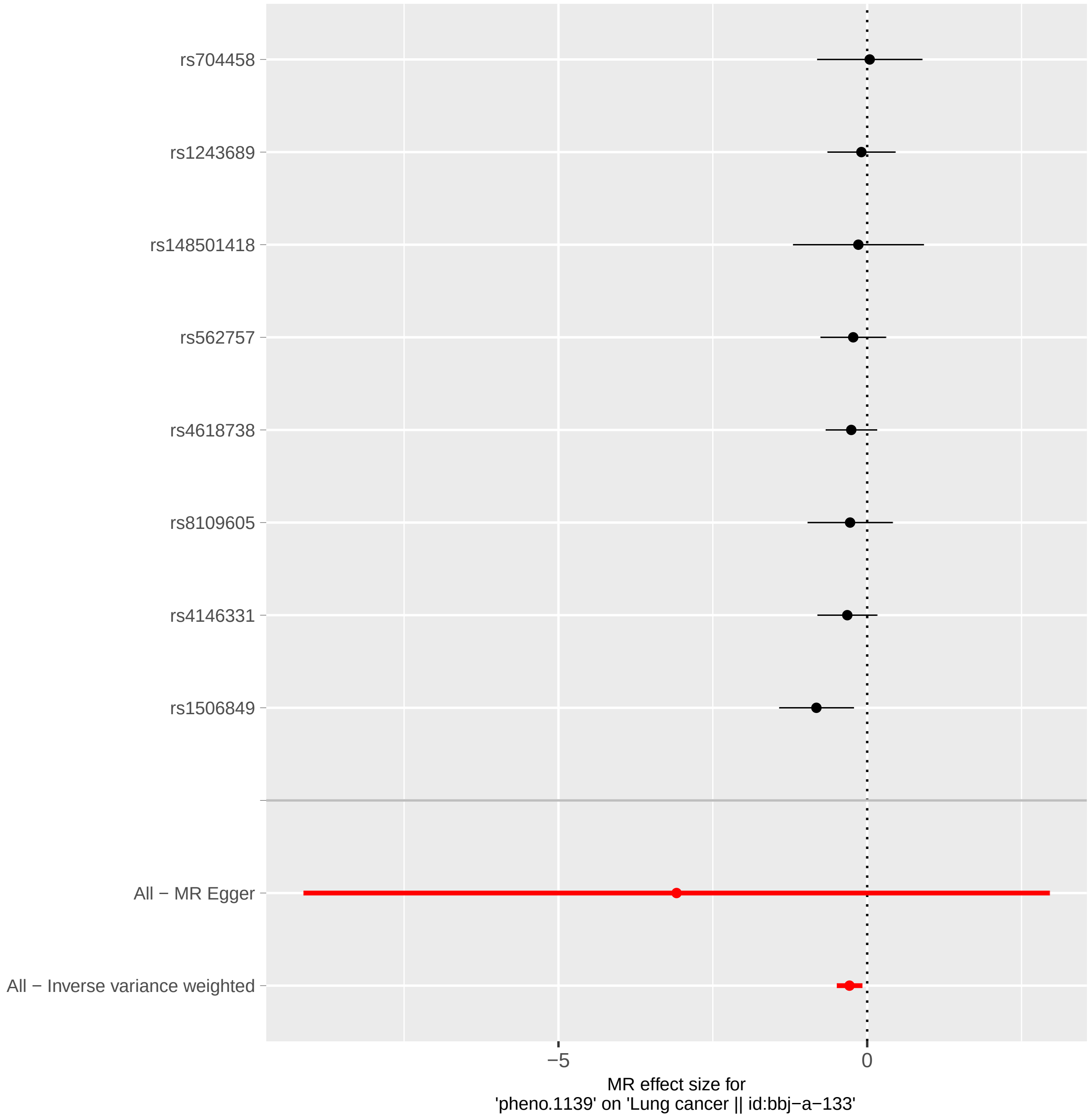

rs704458
rs1243689
rs148501418
rs562757
rs4618738
rs8109605
rs4146331
rs1506849
All − MR Egger
All − Inverse variance weighted
−5
0
MR effect size for
'pheno.1139' on 'Lung cancer || id:bbj−a−133'
